# The causal effect of reproductive factors on pelvic floor dysfunction: a Mendelian randomization study

**DOI:** 10.1186/s12905-024-02914-6

**Published:** 2024-01-28

**Authors:** Shufei Zhang, BingShu Li, Jianfeng Liu, Lian Yang, Hanyue Li, Li Hong

**Affiliations:** Department of Obstetrics and Gynecology, Renmin Hospital of Wuhan University, Wuhan, Hubei Province 430060 China

**Keywords:** Pelvic floor dysfunction, Female genital prolapse, Stress urinary incontinence, Mendelian randomization, Reproductive factors

## Abstract

**Background:**

Pelvic floor dysfunction (PFD) is an extremely widespread urogynecologic disorder, the prevalence of which increases with aging. PFD has severely affected women’s quality of life and has been called a social cancer. While previous studies have identified risk factors such as vaginal delivery and obesity for PFD, other reproductive factors, including age at menarche (AAMA), have been largely overlooked. Therefore, we used a Mendelian randomization (MR) study for the first time to investigate the potential causal relationship between reproductive factors and PFD.

**Methods:**

We obtained summary statistics from genome-wide association studies (GWAS) for female genital prolapse (FGP), stress urinary incontinence (SUI), and five reproductive factors. Two-sample Mendelian randomization analysis (TSMR) was performed to explore the causal associations between these factors. The causal effects of reproductive factors on FGP and SUI were primarily estimated using the standard inverse variance weighting (IVW) method, with additional complementary and sensitivity analyses conducted using multiple approaches. A multivariate Mendelian randomization (MVMR) study was also conducted to adjust for pleiotropic effects and possible sources of selection bias and to identify independent exposure factors.

**Results:**

Our findings revealed that advanced age at first sexual intercourse (AFS) and age at first birth (AFB) exhibited negative causal effects on both FGP and SUI. AAMA showed negative causal effects solely on FGP, while age at last live birth (ALB) and age at menopause (AAMO) did not demonstrate any causal effect on either FGP or SUI. And the MVMR results showed that AFB and AFS had independent negative causal effects on FGP and SUI, respectively.

**Conclusions:**

This study, for the first time, investigates the causal relationship between reproductive factors and PFD. The results suggested a causal relationship between some reproductive factors, such as AFB and AFS, and PFD, but there were significant differences between FGPand SUI. Therefore, future studies should explore the underlying mechanisms and develop preventive measures for reproductive factors to reduce the disease burden of PFD.

**Supplementary Information:**

The online version contains supplementary material available at 10.1186/s12905-024-02914-6.

## Introduction

Pelvic floor dysfunction (PFD) is the most common urogynecological disorder, with pelvic organ prolapse (POP) and stress urinary incontinence (SUI) being the most prevalent [1]. Epidemiological studies have shown that SUI affects 46% of women, and the lifetime risk of POP surgery ranges from 12 to 19%, with over 3 million POP procedures performed annually in the United States alone, already creating a heavy disease burden [2–4]. PFD arises from pelvic floor support structure laxity, mechanical injury, and other factors, with female genital prolapse (FGP), characterized by the descent of vaginal or uterine tissues into or through the vagina, being the primary clinical manifestation of POP [4]. And SUI is characterized by involuntary urinary leakage when intra-abdominal pressure surpasses urethral pressure [2]. Currently, the diagnosis of PFD primarily relies on symptomatology. Among them, POP is mainly diagnosed using the POP-Q score, a criterion that was proposed in 1996 and is still in use today. This scoring system involves determining the relative positions of six key points through physical examination, resulting in a score that categorizes POP into four stages [5]. For mild PFD, patients are typically recommended to undergo pelvic floor muscle exercises or electrical stimulation. On the other hand, for severe PFD, relevant surgical procedures are usually recommended. The most commonly performed procedure for POP is pelvic floor reconstruction surgery, while vaginal tension-free midurethral suspension is considered the gold standard procedure for SUI [1]. Therefore, it is crucial to delay or prevent the progression of mild PFD.

Vaginal delivery is recognized as the strongest risk factor for PFD in women under 60 years of age, and obesity and previous hysterectomy have also been identified as risk factors [6–8]. While some reproductive factors, such as pregnancy and childbirth, have established associations with PFD, others, including age at menarche (AAMA), age at menopause (AAMO), age at first intercourse (AFS), age at first birth (AFB), and age at last live birth (ALB), have received less attention [4, 9]. Therefore, this study focuses on the prevalent FGP and SUI as representatives of PFD and employs a two-sample Mendelian randomization analysis (TSMR) to investigate the potential causal relationship between five reproductive factors (AAMA, AAMO, AFS, AFB, and ALB) and SUI and FGP, and multivariate Mendelian randomization (MVMR) study was used to explore the causal relationships independently. The objective is to bridge the gap in understanding the connection between reproductive factors and PFD development, providing insights into the prevention and management of PFD.

## Materials and methods

### Study design

**Fig. 1:**
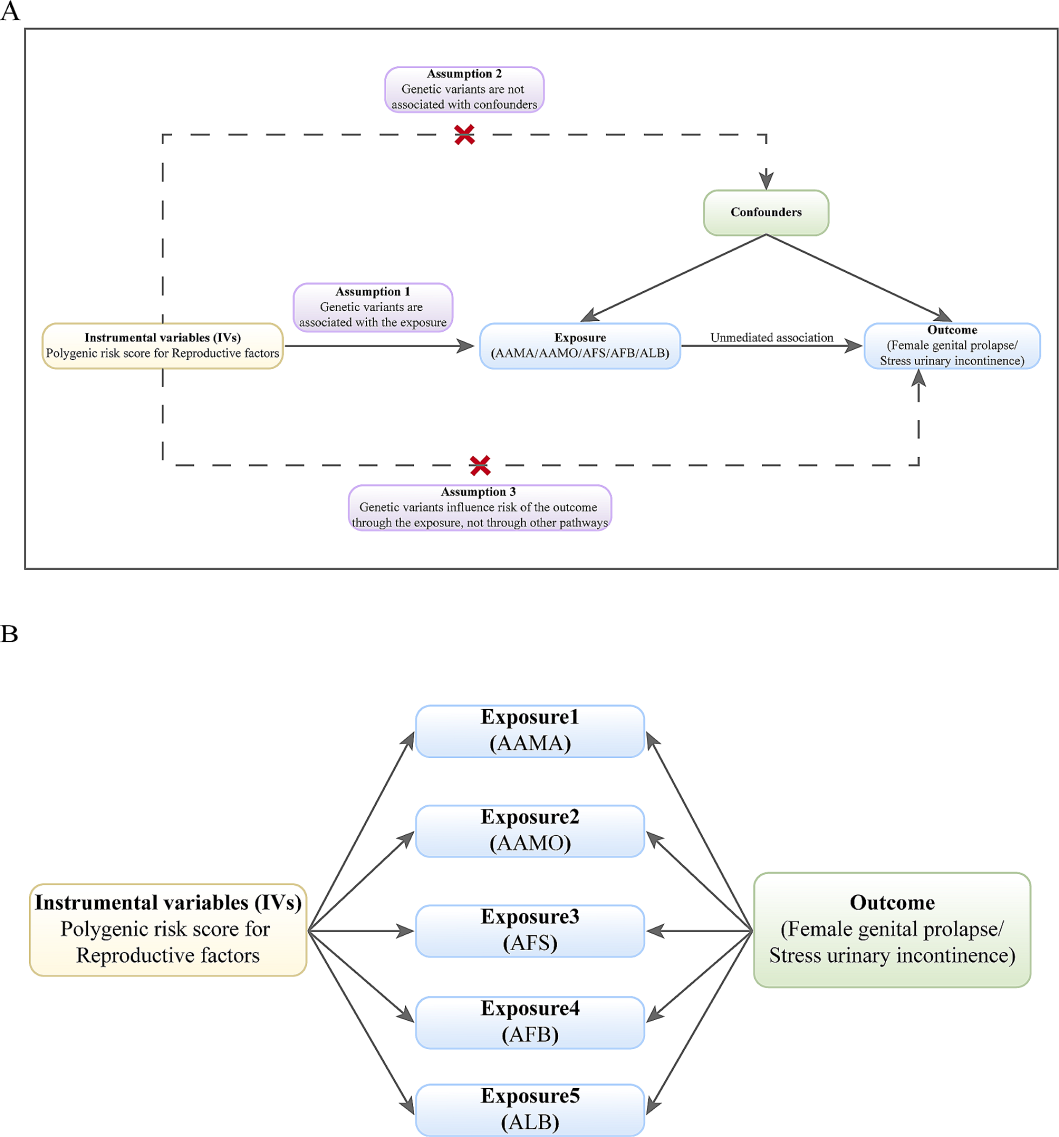
Schematic representation of TSMR and MVMR. **(A)** Schematic representation of TSMR. MR is based on three key assumptions: first, there is a significant association between genetic variation and exposure; second, there is no correlation between the IV and any confounding factors; and third, IV is associated with FGP and SUI (outcome) only through reproductive factors (exposure) and not through direct association. **(B)** Schematic representation of MVMR

This study was performed by TSMR and MVMR with Single nucleotide polymorphisms (SNPs) obtained from the genome-wide association study (GWAS) pooled data (Fig. 1). Reproductive factors were selected as exposures including AAMA, AFS, AFB, ALB, and AAMO, while SUI with FGP was selected as an outcome. Mendelian randomization (MR) has three key assumptions: (1) there is a significant association between genetic variation and exposure; (2) there is no correlation between instrumental variables (IV) and any confounding factors; and (3) exposure is the only way in which genetic variation affects the outcome of interest [10].

### Data sources

Five reproductive factors were included in this study. IV for AAMA was obtained from the GWAS study of Howe LJ and included 7,890,254 SNPs; IVs for AFS and AFB were obtained from the GWAS study of Mills MC et al. and identified 16,359,424 and 10,766,720 SNPs, respectively [11]. IVs for ALB were obtained from the GWAS study of Neale et al. and identified 10,894,596 SNPs. IVs for AAMO were obtained from the GWAS study of Day et al. and identified 2,418,696 SNPs [12]. Data related to SUI were obtained from a GWAS study by Ben Elsworth et al. including 4340 European female cases and 458,670 European pedigree controls, and 9,851,867 SNPs were identified. Diagnosis of SUI based on main ICD10: N39.3. Data related to FGP were obtained from a GWAS study in 2021, including 9092 European female cases and 68,969 European pedigree controls, and 16,377,670 SNPs were identified [13]. Diagnosis of FGP based on main ICD10: N81. It is worth noting that some of the GWAS datasets we used also contained males, which may have influenced our outcome determination to some extent. Therefore, the fact that the reproductive factors we chose were all female-specific and that the FGP case group was all female may circumvent this influence to some extent. All data are available at https://gwas.mrcieu.ac.uk/ and this paper does not contain any identifiable patient information and therefore does not require ethical approval (Table 1).

**Table 1:** The characteristics of GWAS studies on the exposures and outcomes

Exposure	Consortium	Total population	Cases/controls	Ethnicity
Age at menarche	Within family GWAS consortium	29,346	NA	European
Age at first sexual intercourse	NA	397,338	NA	European
Age at first birth	NA	418,758	NA	European
Age at last live birth	Neale Lab	123,676	NA	European
Age at menopause	ReproGen	69,360	NA	European
Outcome	Consortium	Total population	Cases/controls	Ethnicity
Female genital prolapse	NA	78,061	9,092/ 68,969	European
Stress urinary incontinence	MRC-IEU	463,010	4,340/ 458,670	European

### IVs extraction

A threshold of *p* < 5 × 10 − 8 was chosen for the extraction of IVs, and to avoid bias due to linkage disequilibrium (LD), r2 = 0.001 was set, as well as the number of bases (kb > 10,000) between two SNPs [14].

### Statistical analysis

In TSMR, the causal effect of reproductive factors on FGP was estimated mainly by the standard inverse variance weighted (IVW) method, while MR-Egger, weighted median, simple mode, and weight mode methods were also performed as complementary analyses [15]. Cochran’s Q test was subsequently performed to assess heterogeneity, and *P* > 0.05 was considered as no heterogeneity in the included IVs, ignoring the effect of heterogeneity on the estimation of causal effects; if there was significant heterogeneity, random-effects IVW approach was used (*p* < 0.05) [16, 17]. Bias due to horizontal pleiotropy was assessed by the MR-Egger intercept test, and MR-Egger regression analysis and *P* > 0.05 could be considered as a weak possibility of genetic pleiotropy, and its effect was ignored [18]. The reliability of TSMR analysis results was assessed by a leave-one-out test [19]. Finally, considering the importance of five factors for PFD, we further included five factors for MVMR analysis to determine independent exposure. All data analyses were performed in the TwoSampleMR package (R version: 4.2.1) [20, 21]. Differences were considered statistically significant when *P* < 0.05.

## Results

### Causal effect of AAMA and AAMO on FGP

**Fig. 2:**
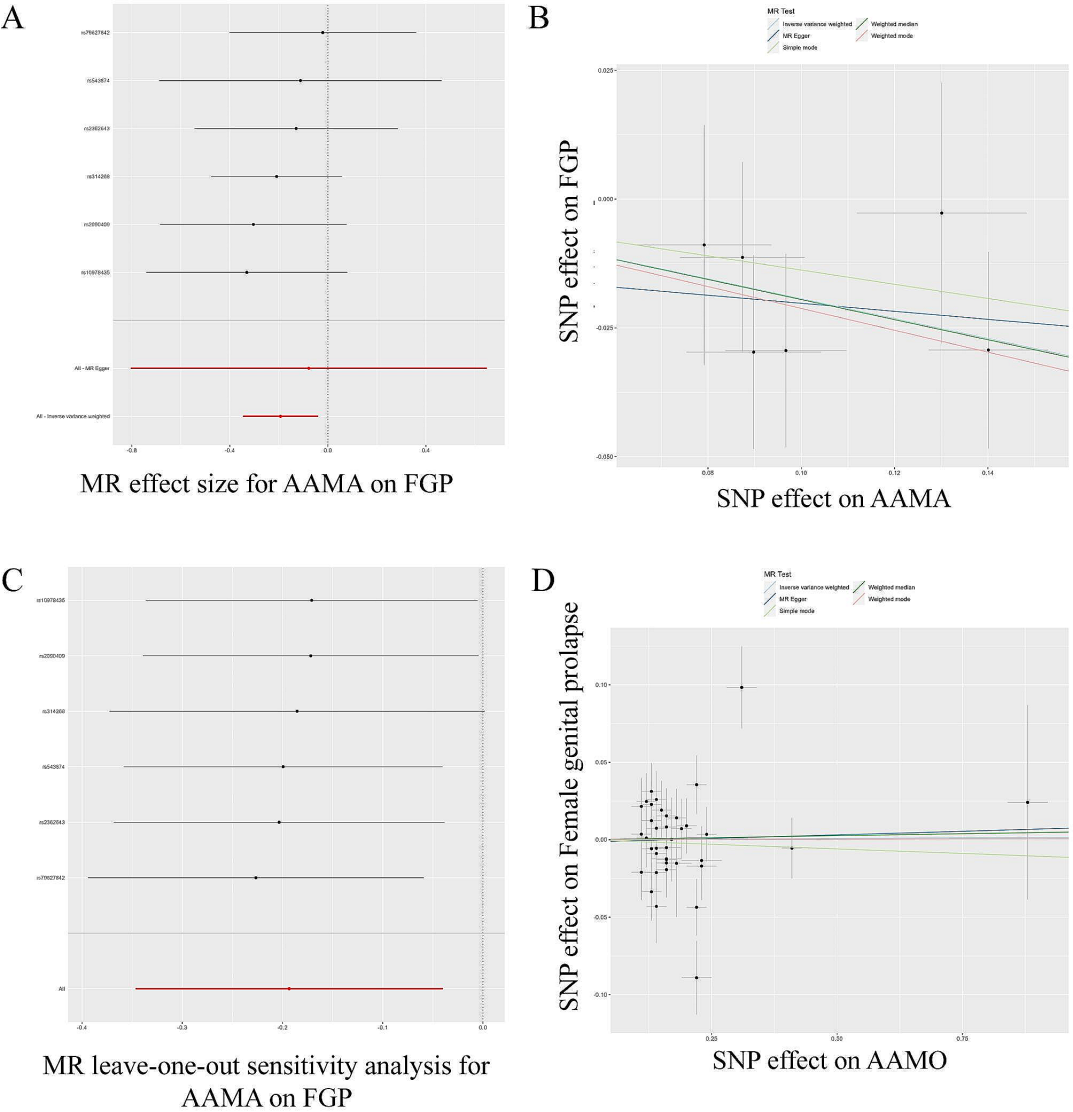
TSMR analysis of AAMA and AAMO with FGP. TSMR of AAMA with FGP **(A)** forest plot; **(B)** dot plot; **(C)** leave-one-out test plot. TSMR of AAMO with FGP **(D)** dot plot

We first assessed the causal effect of AAMA on FGP (Table 2; Fig. 2A-C). The results of the IVW assessment showed a negative causal effect of AAMA on FGP (OR = 0.824, 95% CI: 0.707–0.961; *p* = 0.014). Cochran’s Q test (Cochran’s Q = 1.723; *p* = 0.885) and MR-Egger regression (Egger intercept =-0.012; *p* = 0.766) showed no heterogeneity with horizontal polymorphism, and leave-one-out tests showed reliable and stable results.

We also assessed the causality of AAMO on FGP (Table 2; Fig. 2D). IVW assessment showed a null causality of AAMO on FGP (OR = 1.002, 95% CI: 0.957–1.048; *P* = 0.945). This result was verified by other methods, indicating that there was no causal relationship between AAMO and FGP.

**Table 2:** Two-sample MR estimates of relationship between female reproductive factors and female genital prolapse

Exposure	MR Method	Female genital prolapse			Heterogeneity		Horizontal pleiotropy	
		No. of SNPs	OR (95% CI)	*P*-Value	Cochran’s Q	P-Value	Egger intercept	*P*-Value
Age at menarche	IVW	6	0.824 (0.707–0.961)	0.014	1.723	0.885	-0.012	0.766
	MR-Egger		0.925 (0.447–1.913)	0.844				
	WM		0.823 (0.675–1.004)	0.054				
	Simple mode		0.871 (0.665–1.142)	0.364				
	Weighted mode		0.809 (0.637–1.026)	0.141				
Age at menopause	IVW	38	1.002 (0.957–1.048)	0.945	63.447	0.004	-0.002	0.896
	MR-Egger		1.009 (0.892–1.142)	0.883				
	WM		1.005 (0.950–1.063)	0.857				
	Simple mode		0.988 (0.893–1.094)	0.821				
	Weighted mode		1.001 (0.933–1.073)	0.986				
Age at first birth	IVW	55	0.846 (0.759–0.943)	0.003	115.379	2.392e-06	0.007	0.717
	MR-Egger		0.770 (0.459–1.293)	0.327				
	WM		0.854 (0.758–0.962)	0.010				
	Simple mode		0.738 (0.542–1.005)	0.059				
	Weighted mode		0.762 (0.588–0.989)	0.046				
Age at last live birth	IVW	4	0.621 (0.303–1.272)	0.193	1.670	0.644	0.072	0.445
	MR-Egger		0.038 (0.000–13.374)	0.388				
	WM		0.711 (0.288–1.759)	0.461				
	Simple mode		0.766 (0.216–2.711)	0.707				
	Weighted mode		0.748 (0.216–2.594)	0.678				
Age at first sexual intercourse	IVW	154	0.710 (0.575–0.878)	0.002	161.365	0.306	0.004	0.622
	MR-Egger		0.567 (0.226–1.422)	0.228				
	WM		0.665 (0.487–0.908)	0.010				
	Simple mode		0.650 (0.240–1.763)	0.399				
	Weighted mode		0.706 (0.298–1.670)	0.429				

### Causal effect of AAMA and AAMO on SUI

We assessed the causal effect of AAMA and AAMO on SUI (Table 3 & SUP Fig. 1). The results of the IVW assessment showed that the causal effect of AAMA on SUI was null (OR = 0.999, 95% CI: 0.997–1.001; *P* = 0.462), and other methods verified this result. Also, the results of the IVW assessment showed a null causality of AAMO on SUI (OR = 1.000, 95% CI: 0.999–1.001; *P* = 0.484), which was verified by other methods. It indicates that there is no causal effect of either AAMA or AAMO on SUI.

### Causal effect of AFB and ALB on FGP

**Fig. 3:**
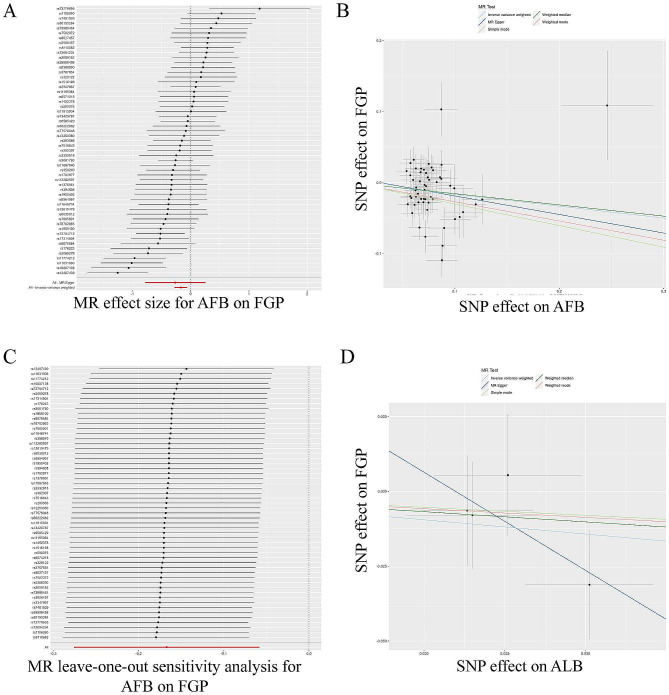
TSMR analysis of AFB and ALB with FGP. TSMR of AFB with FGP **(A)** forest plot; **(B)** point plot; **(C)** leave-one-out test. TSMR of ALB with FGP **(D)** point plot

Next, we evaluated the causal effect of AFB on FGP (Table 2; Fig. 3A-C). The results of the IVW evaluation showed a negative causal effect of AFB on FGP (OR = 0.846, 95% CI: 0.759–0.943; *p* = 0.003). The WM (OR = 0.854, 95% CI: 0.758–0.962; *p* = 0.010), and weighted model (OR = 0.762, 95% CI: 0.588–0.989; *p* = 0.046) also validated this result. Heterogeneity was demonstrated by Cochran’s Q test (Cochran’s Q = 115.379; *p* = 2.392e-06) and MR-Egger regression (Egger intercept = 0.007; *p* = 0.717), but not horizontal polymorphism, and the leave-one-out test showed reliable and stable. After applying the random-effects IVW model, the same results are obtained still the same and the results are presented as forest plots. We then further explored the causal relationship between ALB and FGP (Table 2; Fig. 3D). The causal relationship assessed by IVW showed a null causal relationship between ALB and FGP (OR = 0.621, 95% CI: 0.303–1.272; *P* = 0.193), and this result was verified by other methods, indicating that no There is no causal relationship between ALB and FGP.

### Causal effect of AFB and ALB on SUI

**Fig. 4:**
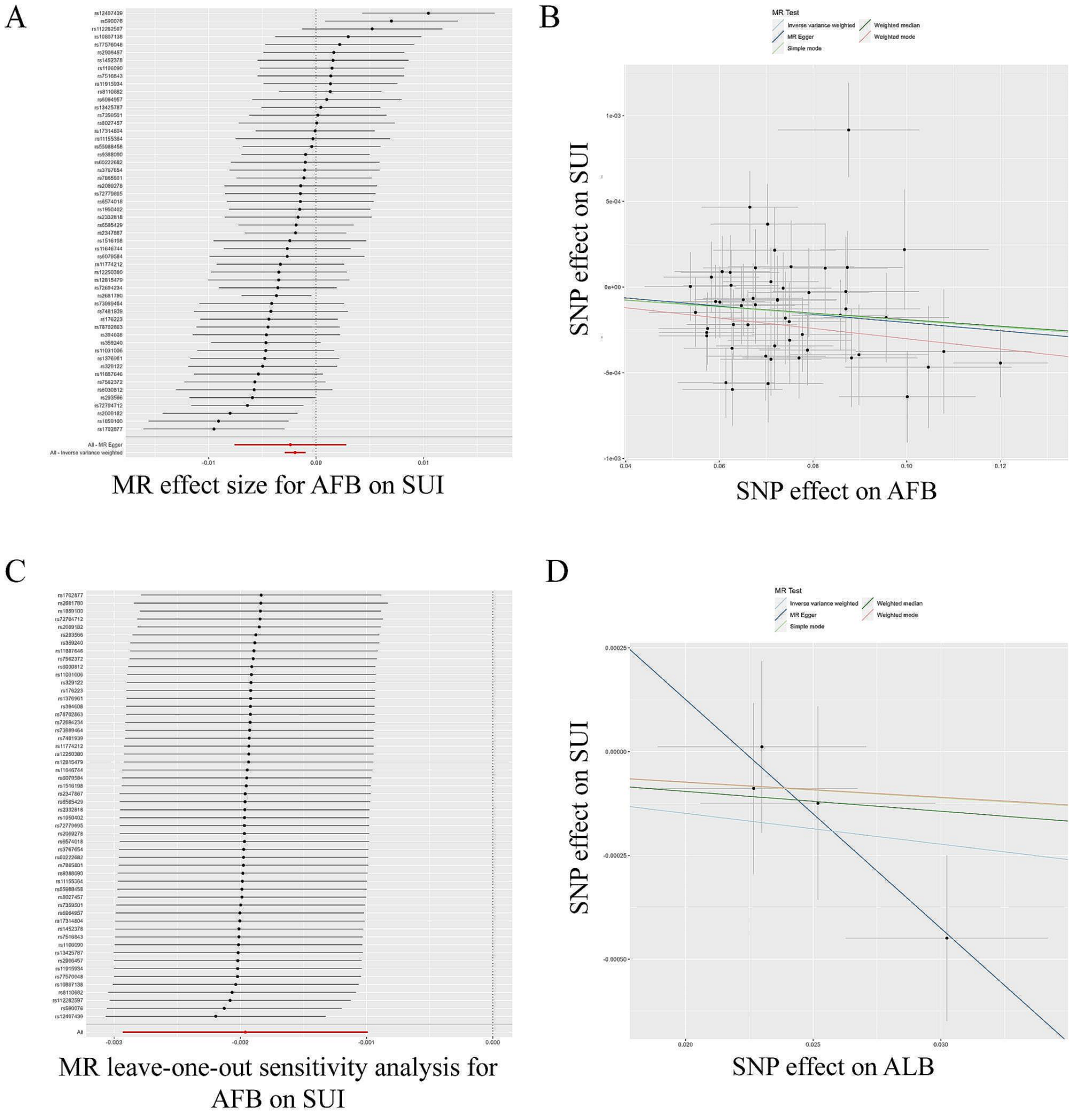
TSMR analysis of AFB and ALB with SUI. TSMR of AFB with SUI **(A)** forest plot; **(B)** point plot; **(C)** leave-one-out test plot. TSMR of ALB with SUI **(D)** point plot

Then, we evaluated the causal effect of AFB on SUI (Table 3; Fig. 4A-C). The results of the IVW evaluation showed that AFB had a negative causal effect on SUI (OR = 0.998, 95% CI: 0.997–0.999; *P* = 7.659e-05). The WM (OR = 0.998, 95% CI: 0.997–0.999; *p* = 0.004), and weighted model (OR = 0.997, 95% CI: 0.994–0.999; *p* = 0.032) also validated this result. Heterogeneity was demonstrated by Cochran’s Q test (Cochran’s Q = 71.058; *p* = 0.049) and MR-Egger regression (Egger intercept = 3.223e-05; *p* = 0.869), but not horizontal polymorphism, and the leave-one-out test showed reliable and stable results. The same analysis was performed using the random-effects IVW model, and the same conclusions were obtained, with the results presented as a forest plot. We then further explored the causal relationship between ALB and SUI (Table 3; Fig. 4D). The results of the IVW assessment showed a null causality of ALB on SUI (OR = 0.993, 95% CI: 0.985–1.001; *P* = 0.071), other methods also validated this result, indicating that there is no causality.

**Table 3:** Two-sample MR estimates of relationship between female reproductive factors and Stress urinary incontinence

Exposure	MR Method	Female genital prolapse			Heterogeneity		Horizontal pleiotropy	
		No. of SNPs	OR (95% CI)	*P*-Value	Cochran’s Q	*P*-Value	Egger intercept	*P*-Value
Age at menarche	IVW	5	0.999 (0.997–1.001)	0.462	4.057	0.398	1.069e-04	0.856
	MR-Egger		0.998 (0.989–1.008)	0.760				
	WM		1.000 (0.997–1.002)	0.779				
	Simple mode		1.001 (0.997–1.005)	0.613				
	Weighted mode		1.001 (0.998–1.004)	0.554				
Age at menopause	IVW	37	1.000 (0.999–1.001)	0.484	70.584	0.501e-03	1.287e-04	0.418
	MR-Egger		0.999 (0.998–1.001)	0.588				
	WM		1.000 (0.999–1.001)	0.365				
	Simple mode		1.001 (0.999–1.002)	0.113				
	Weighted mode		1.001 (1.000–1.001)	0.189				
Age at first birth	IVW	54	0.998 (0.997–0.999)	7.659e-05	71.058	0.049	3.223e-05	0.869
	MR-Egger		0.998 (0.992–1.003)	0.372				
	WM		0.998 (0.997–0.999)	0.004				
	Simple mode		0.998 (0.995–1.001)	0.189				
	Weighted mode		0.997 (0.994–0.999)	0.032				
Age at last live birth	IVW	4	0.993 (0.985–1.001)	0.071	2.268	0.519	0.001	0.286
	MR-Egger		0.946 (0.886–1.010)	0.240				
	WM		0.995 (0.985–1.001)	0.358				
	Simple mode		0.996 (0.980–1.013)	0.683				
	Weighted mode		0.996 (0.982–1.011)	0.655				
Age at first sexual intercourse	IVW	145	0.994 (0.991–0.997)	8.716e-05	216.998	8.082e-05	1.427e-04	0.178
	MR-Egger		0.985 (0.972–0.999)	0.035				
	WM		0.994 (0.990–0.997)	4.820e-04				
	Simple mode		0.996 (0.985–1.007)	0.418				
	Weighted mode		0.994 (0.985–1.004)	0.224				

### Causal effect of AFS on FGP and SUI

**Fig. 5:**
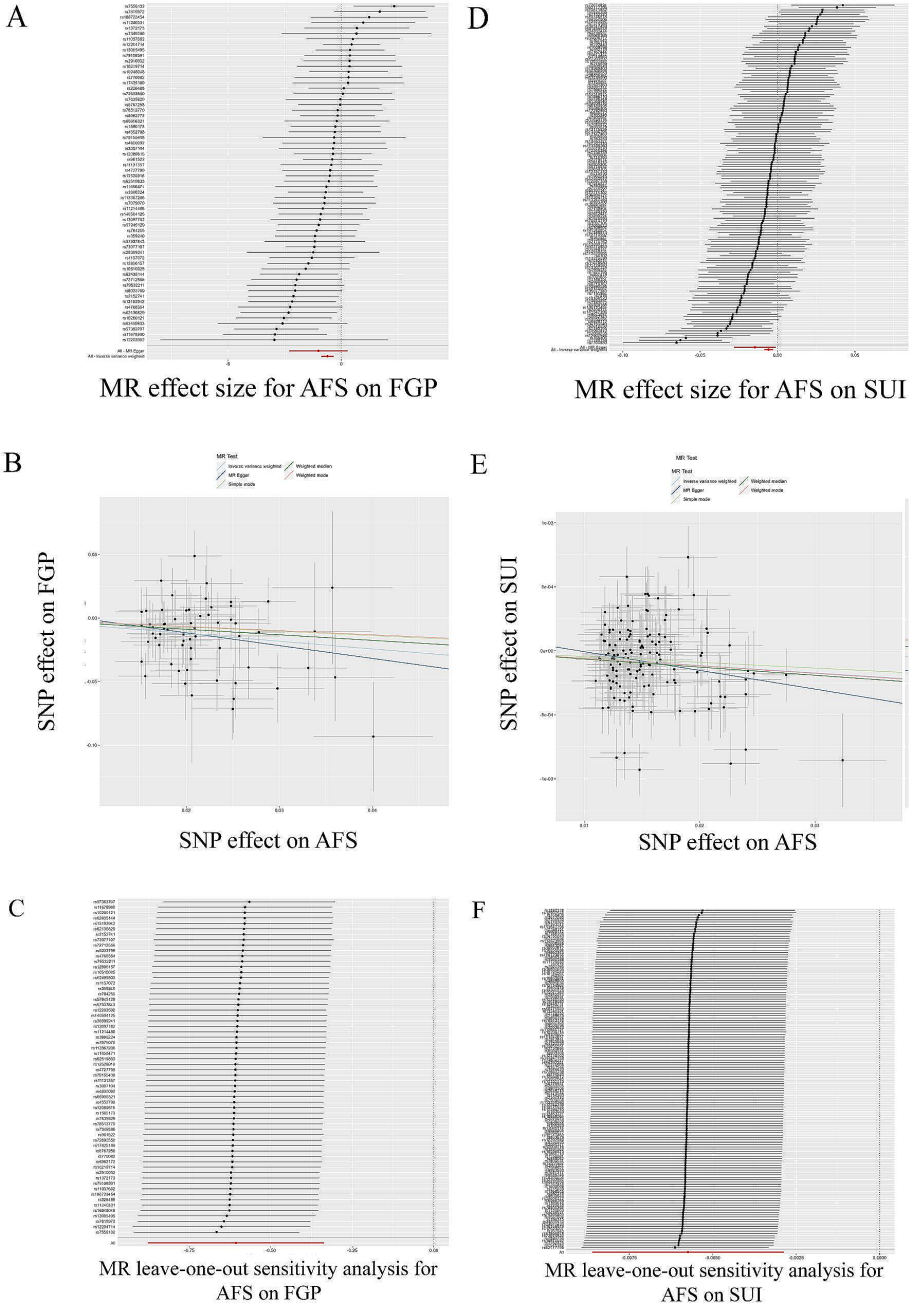
TSMR analysis of AFS with FGP and SUI. TSMR of AFS with FGP **(A)** forest plot; **(B)** dot plot; **(C)** leave-one-out test plot. TSMR of AFS with SUI **(D)** forest plot; **(E)** dot plot; **(F)** leave-one-out test plot

Furthermore, we evaluated the causal effect of AFS on FGP and SUI (Tables 2 and 3; Fig. 5). The causal relationship assessed with IVW showed a negative causal effect of AFS on FGP (OR = 0.710, 95% CI: 0.575–0.878; *p* = 0.002), and similar results were obtained with WM (OR = 0.665, 95% CI: 0.487–0.908; *p* = 0.010) analysis. Cochran’s Q test (Cochran’s Q = 161.365; *p* = 0.306) and MR-Egger regression (Egger intercept = 0.004; *p* = 0.622) showed no heterogeneity with horizontal polymorphism, and the leave-one-out test showed reliable and stable results.

Similar to the above, the causality assessed by IVW showed a negative causality of AFS on SUI as well (OR = 0.994, 95% CI: 0.991–0.997; *p* = 8.716e-05), using MR-Egger (OR = 0.985, 95% CI: 0.972–0.999; *p* = 0.035) and WM (OR = 0.994, 95% CI: 0.990–0.997; *p* = 4.820e-04) analyses yielded similar results. Cochran’s Q test (Cochran’s Q = 216.998; *p* = 8.082e-05) and MR-Egger regression (Egger intercept = 1.427e-04; *p* = 0.178) showed the presence of heterogeneity but not horizontal polymorphism, and the leave-one-out test showed reliable and stable results, and as above, we can assume that there is also a negative causality of AFS on SUI i.e., an increase in AFS can reduce the risk of SUI.

### MVMR analysis of reproductive factors on PFD

**Fig. 6:**
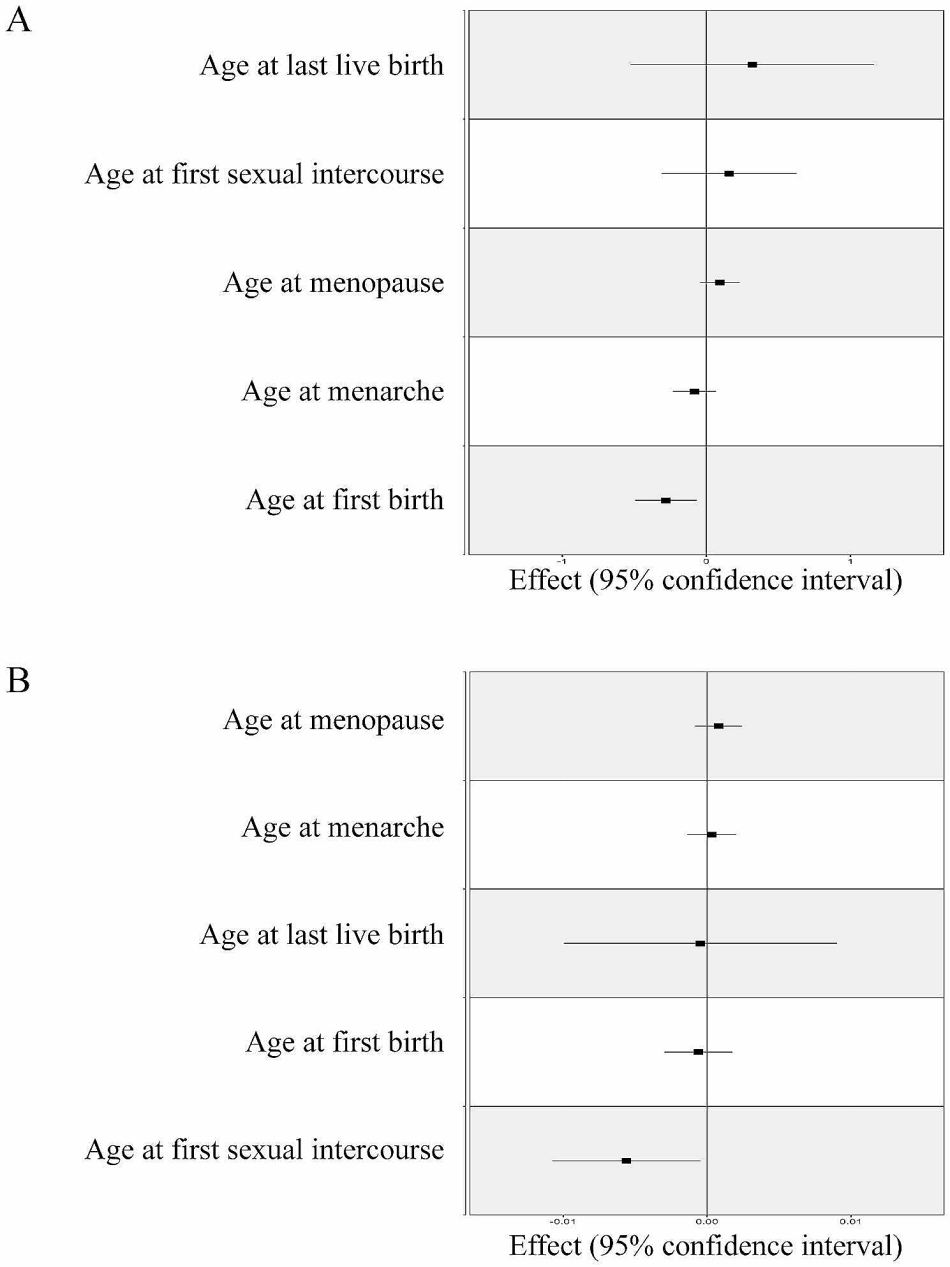
MVMR analysis of female reproductive factors and PFD. **(A)** MVMR analysis of female reproductive factors with FGP; **(B)** MVMR analysis of female reproductive factors with SUI

Finally, considering the importance of the five reproductive factors for PFD, we also performed MVMR analysis to reduce the effect of confounding and to identify independent exposures (Table 4; Fig. 6). We observed a negative direct causal effect of AFB on FGP (OR = 0.756, 95% CI: 0.610–0.936; *p* = 0.010); and a negative direct causal effect of AFS on SUI (OR = 0.994, 95% CI: 0.989–0.999; *p* = 0.032).

**Table 4:** MVMR estimates of relationship between female reproductive factors and Pelvic floor dysfunction

Exposure	MR Method	Female genital prolapse			Stress urinary incontinence		
		No. of SNPs	OR (95% CI)	*P*-Value	No. of SNPs	OR (95% CI)	*P*-Value
Age at menarche	IVW	3	0.920 (0.793–1.069)	0.276	3	1.000 (0.999–1.002)	0.706
Age at menopause		67	1.098 (0.958–1.258)	0.180	59	1.001 (0.999–1.002)	0.339
Age at first birth		29	0.756 (0.610–0.936)	0.010	28	0.999 (0.997–1.002)	0.620
Age at last live birth		2	1.376 (0.591–3.204)	0.460	2	1.000 (0.990–1.009)	0.923
Age at first sexual intercourse		101	1.171 (0.733–1.872)	0.509	97	0.994 (0.989–0.999)	0.032

## Discussion

Menarche, the onset of the first menstrual period, is a significant event in a woman’s life. Previous studies have demonstrated associations between early AAMA and conditions such as breast and endometrial cancer, which are often attributed to hormonal exposure and menstrual cycling [22]. Additionally, AAMA has been linked to high body mass index, diabetes, and cardiovascular diseases [23–25].

Interestingly, earlier menarche has also been associated with negative effects on pelvic floor biomechanical properties and female genitalia, contributing to the development of PFD [26]. Our findings confirm this association, revealing a negative causal relationship between AAMA and FGP. However, it is worth noting that the interval between AAMA and AFB may be a more accurate assessment criterion. Later menarche could potentially reduce the impact of hormonal exposure on pelvic floor biomechanical properties by shortening the time between menarche and AFB, thereby lowering the risk of developing FGP [27]. The influence of AAMA on PFD development may not solely be attributed to long-term hormone exposure but also to psychosocial and other factors. Precocious puberty may lead to earlier sexual activity, increasing the likelihood of risk factors such as early pregnancy and indirectly raising the risk of FGP.

Unlike the negative causal relationship observed between AAMA and FGP, our study found no significant causal relationship between AAMA and SUI. Some reports have indicated that up to 54% of women with POP also experience SUI. However, the relationship between POP and SUI remains inconclusive, as apical and anterior prolapse can mask SUI symptoms, which may manifest only after prolapse surgery [28]. AAMA may contribute to prolapse symptoms earlier, potentially masking mild SUI. Further high-quality clinical studies are needed to establish the relationship [29]. Our study also found no significant causal relationship between AAMO and both FGP and SUI. This result may be due to the fact that most of the onset of PFD is caused by pregnancy- and childbirth-related injuries and that the weight of AAMO is insufficient to significantly impact the development of PFD.

Our results suggested a negative causal effect of AFS on both FGP and SUI, and the MVMR results showed that AFS was independently associated with the risk of SUI. Previous studies have shown that in cohorts of women who have undergone vaginal delivery, younger age at first delivery is protective against later SUI and POP procedures. However, these results conflict with the notion that younger age at first delivery is a potential risk factor for later POP [30]. In contrast, our study revealed a negative causal effect of AFB on FGP and SUI, and AFB was even more independently associated with the risk of FGP, consistent with the latter perspective. It is worth noting that the previous study focused on surgical outcomes, while our study examined morbidity, including patients with mild to moderate PFD who often receive conservative treatment instead of undergoing surgery. This difference may account for the divergent findings. And the two MVMR results are not consistent may suggested to us that although FGP and SUI belong to the same PFD, the underlying mechanism may be different, and SUI may be related to early sexual activity while FGP may be more attributable to pregnancy and delivery, which of course needs to be corroborated by more studies.

While no significant causal relationship was observed between ALB and FGP or SUI, there was a slight negative correlation. This observation can be explained by the fact that PFD primarily results from pelvic floor structural damage following the initial vaginal delivery [31]. An earlier sexually active period may be associated with a lack of sexual knowledge or unmet contraceptive needs in some young women, increasing the risk of early pregnancy and the number of pregnancies, which in turn contributes to the development of PFD [32, 33]. Interestingly, genetic correlations between reproductive factors were observed in an MR analysis, particularly between early reproductive factors such as AAMA and AFS, AFB [34]. Another study also demonstrated that earlier AFS was associated with earlier AFB and lower educational attainment, further supporting our aforementioned inference [35].

A study on reproductive life expectancy in US women revealed a decreasing trend in the mean age at menarche (from 13.5 to 12.7 years) and an increased mean reproductive life expectancy (from 35.0 to 37.1 years) over the last six decades [36]. Considering our findings, the declining age at menarche may lead to an elevated risk of developing PFD in the future. Therefore, it is crucial to prioritize early sex education to mitigate the adverse effects of early sexual intercourse and childbirth and enhance awareness of reproductive factors and pelvic floor dysfunction disorders.

This study has several strengths. Firstly, it is the pioneering research to specifically investigate the causal relationship between reproductive factors and PFD in women, signifying a significant advancement in the field. Secondly, all the analyzed datasets exclusively comprise individuals of European descent, thereby potentially reducing the influence of population stratification on the observed association. Nonetheless, there exist several limitations to our study. Due to the limited availability of data regarding PFD and reproductive factors, we obtained GWAS data from the UK Biobank, resulting in some overlap within the study population. This might have introduced a degree of bias. To further validate our findings, future studies should encompass diverse populations. Moreover, it is noteworthy that our dataset comprises both male and female subjects, precluding a separate analysis focused exclusively on female PFD patients. Subsequent research endeavors should strive to incorporate gender-specific data to facilitate subsequent gender-stratified investigations.

Our study addresses a longstanding research gap in the field of PFD by focusing on female reproductive factors. First, researchers need to be encouraged not only to conduct larger-scale GWAS studies, but also to conduct GWAS studies on populations of different economic levels, regions, and ethnicities. There is also a need to focus on the gap between less developed and developed countries and to fill the gaps in relevant research in less developed countries. Furthermore, since female reproductive factors are specific to women and play a crucial role in women’s health, it is essential to conduct more research to explore their correlation with various diseases. This will not only guide clinical practice but also contribute to improving the overall quality of life and health for women.

## Conclusion

Our study establishes a negative causal relationship between AAMA, AFS, and AFB with FGP, while no causal relationship was found between ALB or AAMO and FGP. Similarly, AFS and AFB exhibited negative causal effects on SUI, while AAMA, AAMO, and ALB had no causal relationship with SUI. More importantly, MVMR analysis showed that AFB was independently associated with the risk of FGP and AFS was independently associated with the risk of SUI. In conclusion, our study provides valuable insights for PFD research in women, and future studies should explore underlying mechanisms and implement measures targeting reproductive factors to prevent PFD.

### Electronic supplementary material

Below is the link to the electronic supplementary material.

Supplementary Material 1

## Data Availability

No datasets were generated or analysed during the current study.

## Abbreviations

PFD: Pelvic floor dysfunction
FGP: Female genital prolapse
SUI: Stress urinary incontinence
MR: Mendelian randomization
TSMR: Two-sample Mendelian randomization
MVMR: Multivariate Mendelian randomization
AAMA: Age at menarche
AAMO: Age at menopause
AFS: Age at first intercourse
AFB: Age at first birth
ALB: Age at last live birth
GWAS: Genome-wide association studies
IVW: Standard inverse variance weighting
LD: Linkage disequilibrium
SNPs: Single nucleotide polymorphisms
IV: Instrumental variables
