# Supplementary material for: The causal effect of reproductive factors on pelvic floor dysfunction: a Mendelian randomization study
Source: BMC Womens Health. 2024 Jan 28;24:74. doi: 10.1186/s12905-024-02914-6 (PMC10822177; doi:10.1186/s12905-024-02914-6)
Supplement: Supplementary file 1 — Supplementary Material 1 [file 12905_2024_2914_MOESM1_ESM.docx]

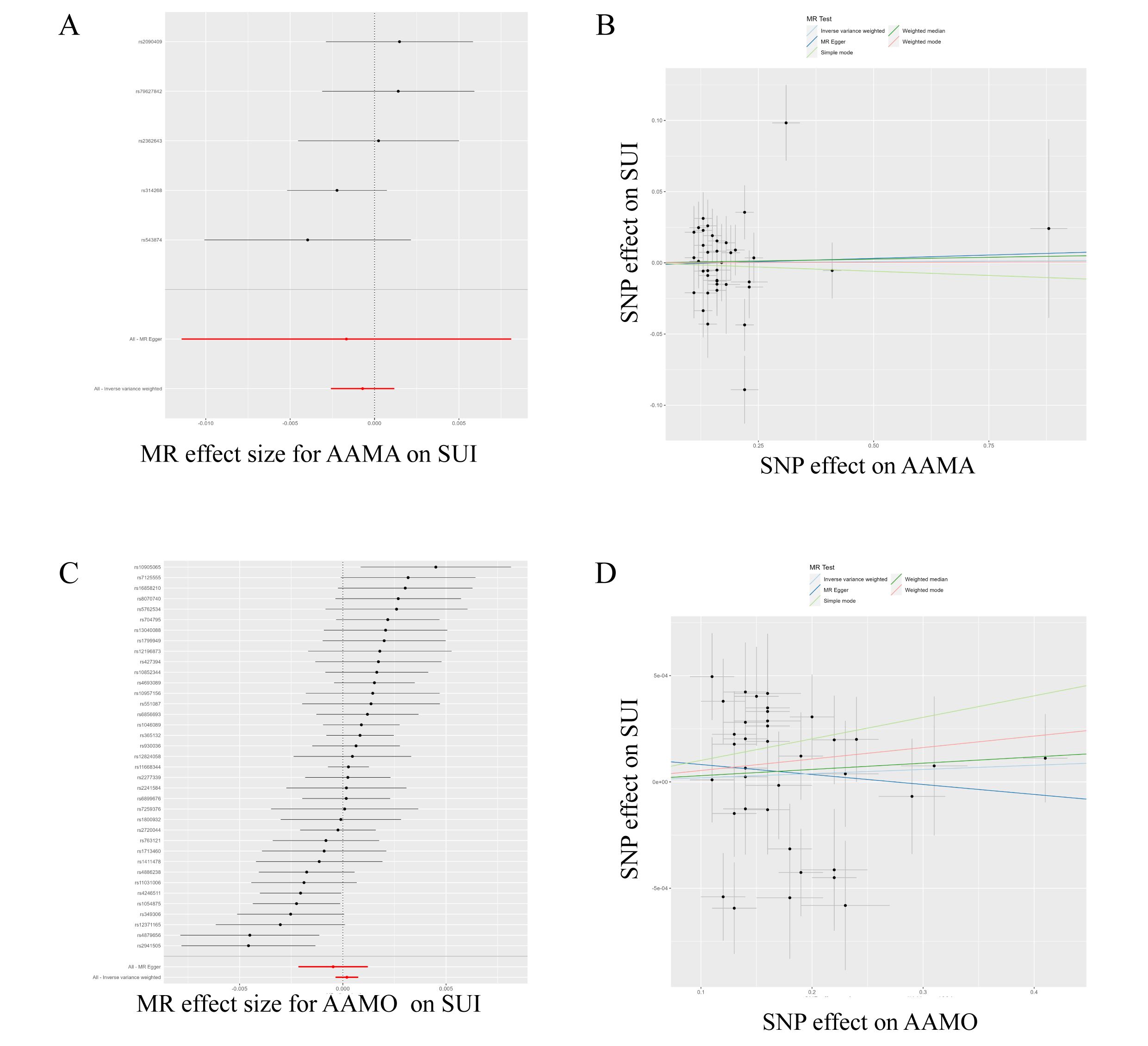


**Sup Figure 1:** TSMR analysis of AAMA and AAMO with SUI. TSMR of AAMA with SUI (A) forest plot; (B) dot plot. TSMR of AAMO with SUI (C) forest plot; (D) dot plot.
